# Abscess of adrenal gland caused by disseminated subacute Nocardia farcinica pneumonia. A case report and mini-review of the literature

**DOI:** 10.1186/1471-2334-9-194

**Published:** 2009-12-02

**Authors:** Michael Tachezy, Philipp Simon, Corina Ilchmann, Yogesh K Vashist, Jakob R Izbicki, Karim A Gawad

**Affiliations:** 1 Department of General-, Visceral- and Thoracic Surgery, University Medical Center Hamburg-Eppendorf, Germany, Martinistraße 52, 20246 Hamburg, Germany; 2 Diagnostic and Interventional Radiology Department and Clinic, University Medical Center Hamburg-Eppendorf, Germany, Martinistraße 52, 20246 Hamburg, Germany; 3 Department of Medical Microbiology, Virology and Hygiene, University Medical Center Hamburg-Eppendorf, Germany, Martinistraße 52, 20246 Hamburg, Germany

## Abstract

**Background:**

Infections caused by *Nocardia farcinica *are uncommon and show a great variety of clinical manifestations in immunocompetent and immunocompromised patients. Because of its unspecific symptoms and tendency to disseminate it may mimic the clinical symptoms and radiologic findings of a tumour disease and the diagnosis of nocardiosis can easily be missed, because there are no characteristic symptoms.

**Case presentation:**

We present a case of an adrenal gland abscess caused by subacute disseminated *N. farcinica *pneumonia.

**Conclusion:**

An infection with *N. farcinica *is potentially lethal because of its tendency to disseminate -particularly in the brain- and its high resistance to antibiotics. Awareness of this differential diagnosis allows early and appropriate treatment to be administered.

## Background

*Nocardia species *are aerobic, gram positive, filamentous, weakly acid-fast bacteria which grow worldwide in soils as well as animal tissues. There are more than eighty *N. species *described until now [1], thereof one-half are recognized as pathogens in humans or animals [2,3]. In recent years because of the use of molecular techniques the taxonomy of *Nocardia *has been revised extensively [2-5].

An infection with *N. farcinica *is potentially lethal because of its tendency to disseminate and its resistance to antibiotics [6-9]. *N. farcinica *is one of the prevalent species causing nocardiosis [10].

Definite rates of pulmonary *N. farcinica *infections are not known, but might not be as rare as generally assumed. Some reports indicate an increasing incidence because of a higher rate of immunodeficient patients, as well as improved techniques to identify the bacteria [11].

Most of the symptomatic patients with pulmonary nocardiosis have a predisposing immunocomprimising disease such as malignancies, advanced HIV infection, diabetes mellitus, renal dysfunction, collagen vascular diseases, alcoholism, tuberculosis, preceding operations, chronic obstructive pulmonary disease (COPD), trauma or abnormal phagocytic activity and medical immunosupression e.g. steroid therapy [3]. In 15% of the cases it occurs without underlying illness [6,12].

The patient presented in this case had no obvious immunodeficiency. Possibly the combination of an alcoholic toxicomania in the past (without any signs of liver disease) and the decreased general condition and nutritional status have made the patient susceptible for the infection.

An extensive Japanese study showed that most of the patients are between 60 and 80 years old [12] and some authors state, that it occurs three times more often in men than in women [6,13,14].

Nocardiosis is usually acquired through the lung, but also by inoculation through traumatic injury of the integument [6,13,15]. Although health care-associated transmission has been documented, human-to-human transmission of nocardiosis is considered to be improbable [2,3,16]. It possibly metastasizes hematogenously into distant organs, especially the brain, followed by kidney, joints, bones and eyes [6,17]. Involvement of other organs like adrenal gland is less common [18-21].

Nocardiosis is a microbiological diagnosis. *N. farcinica *can be isolated in clinical specimen like pus, sputum, bronchial secretion, biopsies, blood and urine in many culture media after two to 14 days [22]. Because of the potentially long culture time it is very important, that the physicians notify the laboratory when *Nocardia *is suspected in a clinical specimen. One of the most important findings is the presentation of the typical morphology of *N. species *surrounded by acute inflammatory cells on a direct Gram-stained smear [2]. Determination of the identification of the *N. species *via biochemical and/or molecular methods (which is the most reliable method for identification) and of course the antimicrobial susceptibility profile is important for the treatment of the patient [23].

When pulmonary nocardiosis is diagnosed, a CT or MRT of the brain and abdomen should be considered.

Therapy of the disease depends -beside the species and its antibiogram- on the severity of the infection, if and where it disseminated and the immune status of the patient.

The empiric gold standard of medical treatment is trimethoprim-sulfamethoxazole (TMP-SMX), which well penetrates the cerebral barrier [24], but *N. farcinica *has a high rate of resistance against TMP-SMX [6-9].

Amikacin, imipenem, third generation cephalosporins, minocycline, netilmicin and amoxicillin-clavulanic acid are second line antibiotics for nocardiosis [3]. There is a characteristic resistance of *N. farcinica *to ampicillin, broad-spectrum cephalosporine, clarithromycin and aminoglycosides except amikacin. It is susceptible to ciprofloxacin, linezolid and imipenem [2].

Patients with disseminated disease, especially the CNS, should be treated with a combined therapy including TMP-SMX and a bactericidal agent or a combination of imipenem and amikacin. Involvement of CNS might be treated with third generation cephalosporins [22]. Further medical treatment would be based on susceptibility results. Therapy must start intravenously and can later be replaced by oral therapy depending on clinical and radiological responses. An alternative antibiotic drug for resistant bacteria is linezolid [3].

The therapy has to be continued for several months because of high relapse rates, depending on the immune status of the patient. If CNS is involved, therapy must last at least 12 months [14,25], followed by monitoring for at least another year after completion of the therapy [26]. Patients with a persisting immunodeficiency should receive a prolonged therapy and a low-dose prophylaxis [27].

In addition to drug treatment a larger abdominal abscess should be treated by drainage and/or radical excision. Cerebral abscesses can be excised and this seems to lower the mortality of the patient compared to solitary drug treatment with or without aspiration/drainage, depending on the size and growing behaviour over time [28].

Data about the prognosis are highly oscillating, mortality rates between 14% and 40% have been published, in case of dissemination even 100% [3]. Depending on the point of diagnosis, the beginning of the treatment and the resistance to antibiotics, as well as secondary host factors, the mortality rate of cerebral abscesses may even be higher, 75% to 90% [29,30].

This case report presents the surprising diagnosis of an adrenal abscess caused by *Nocardia farcinica *mimicking a malignant adrenal mass and nicely demonstrates that pulmonary Nocardia infections can easily be misdiagnosed, because in the beginning there are only uncharacteristic symptoms of acute, subacute or chronic pneumonia. Furthermore, the case illustrates the difficulty to differentiate between adrenal abscess, adrenal metastasis, necrotic malignant tumour and complex adrenal cysts on CT [19], although contrast CT is generally accepted as the cornerstone of adrenal imaging [18].

## Case presentation

Initial reason for hospitalization of the 71 year old female patient in the department of internal medicine was an atypical pneumonia with the symptoms of low-grade fever and a cough without expectoration in combination with a progredient adynamia, a reduced general condition (40%, Karnofsky performance status scale), subtle nausea and a lowered ability to concentrate in the last four weeks. Relevant ancillary diagnoses were arterial hypertension, cardiac insufficiency (NYHA II) and former alcohol addiction without any evidence for a liver disease. Home medication was an antihypertensive combination therapy (Bisoprolol 5 mg and Hydrochlorothiazid/Ramipril 12.5/5 mg).

Physical examination of the underweight patient (47 kg/168 cm) revealed basal crackles on both lungs and low fever (37.8°C). Neurologic status was without pathologic findings.

**Figure 1:**
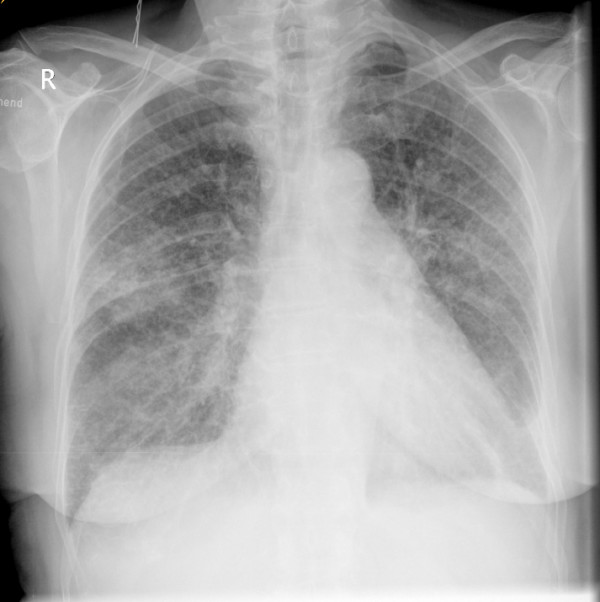
**Thorax x-ray with diffuse small nodules and in some parts also confluent infiltrations; pleural effusion on the left side**.

Pathologic findings in basic diagnostic investigations were a moderate leucocytosis (12.6/nl, normal value 4.0-10.0/nl) and an elevated C-reactive protein (24,65 mg/l, normal value <1.0). Differential count showed the following results: Lymphocytes 12.9% (normal value 25-40%), granulocytes 75.4% (55-75%), basophile granulocytes 0.2% (0.2-1.3%), eosinophile granulocytes 0.2% (0.8-6.2%) monocytes 11.3% (2-8%). Thorax x-ray showed diffuse small nodules and in some parts also confluent infiltrations; pleural effusion on the left side (Figure 1).

A diagnosis of atypical pneumonia was established and an empiric antibiotic treatment with gentamicin and ceftriaxone was initiated. Under therapy febrile temperature and abnormal laboratory values decreased in the following days (Leucocytes 12.1/nl and CrP 7.3 mg/dl).

Initial examination included an abdominal ultrasound, which showed a suspicious right-sided retrohepatic, suprarenal incidentaloma.

**Figure 2:**
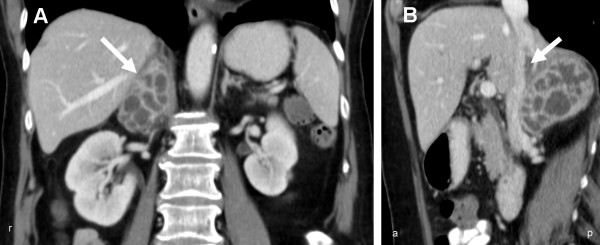
**(A) Contrast-enhanced Coronal CT image of the abdomen shows a 6 cm large suprarenal, contrast enhancing tumour with central septet necrosis (→) (B) Coronal image shows infiltration of the Vena cava inferior and inside a 3-4 cm long thrombus (→)**. In addition an infiltration of the hepatic and renal capsule as well as the diaphragm is shown.

The computed tomography (CT) of abdomen and thorax showed a 6 cm large, central septed tumour in the right adrenal gland with infiltration of the inferior Vena cava and a 3-4 cm long intravenous thrombus inside. In addition, an infiltration of the hepatic and renal capsule as well as the diaphragm was described (Figure 2). These morphologic findings were highly suspicious of a malignant tumour.

Furthermore, CT supported the diagnosis atypical pneumonia with parenchymal infiltrations in the whole lung and partial nodular changes of the parenchyma with reactive effusion. It showed enlarged bronchopulmonary, hilar and mediastinal lymph nodes, which were interpreted as pneumonic inflammatory effects.

Because of the diagnosis of a malignant tumour the patient was transferred to our surgical clinic after recovering from pneumonia. At that time leucocytes and CrP had nearly decreased to a physiologic level (10.4/nl and 2.83 mg/l).

To exclude an endocrine activity of the tumour, levels of suprarenal hormones were determined in plasma and urine, respectively (Metanephrine- and aldosterone-levels in collected urine and dexamethasone suppression tests were at a physiologic level).

We performed a radical in toto resection of the right adrenal gland. The intraoperative finding showed a dense adrenal mass infiltrating the surrounding tissues so that a tangential resection of the Vena cava inferior, diaphragm, retroperitoneum and Gerota's fascia was performed. The specimen was sent for pathological analysis.

A routine chest x-ray in the ICU one day after the operation showed recurrent pneumonic infiltrations. Endotracheal aspirates were purulent and the material was sent for microbiological analysis. An antibiotic treatment was started with piperacillin/sulbactam.

**Figure 3:**
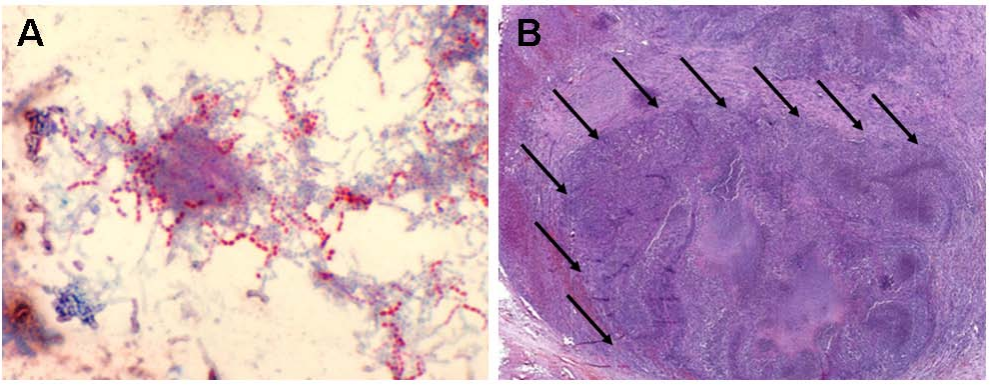
**(A) Cultured *Nocardia farcinica *present as Gram-positive, beaded, thin, branching, Gram-positive rods (Kinyoun-staining, Original magnification ×1000) (B) Histological findings of the adrenal gland were a large necrotic and chronic putrid abscess formation (→) (H&E-staining, Original magnification × 10)**.

On the third postoperative day the patient developed a distal focussed hemiparesis of the left arm, without sensitive deficiencies. On the very same day the preliminary results of microbiological analysis of the culture of bronchioalveolar lavage (on non-selective culture media) were reported as *Nocardia species*. Gram-stained culture has revealed Gram-positive bacilli (Figure 3A). Few days later *N. farcinica *was identified via sequencing of the eubakterial 16s rRNA using polymerase chain reaction (PCR) as described by Weisburg et al[31] Both strands of the ~500 bp PCR product were sequenced using the BigDye Terminator Cycle Sequencing Kit (Applied Biosystems, USA), and analyzed on an ABI PRISM 310 Genetic Analyser (Applied Biosystems, USA). The sequence shows homology over 99,9% to the 16s rRNA gene of *N. farcinica *available at the National Center for Biotechnology Information http://www.ncbi.nlm.nih.gov.

Histology of the adrenal gland revealed a necrotic and chronic putrid abscess-formation (Figure 3B).

**Figure 4:**
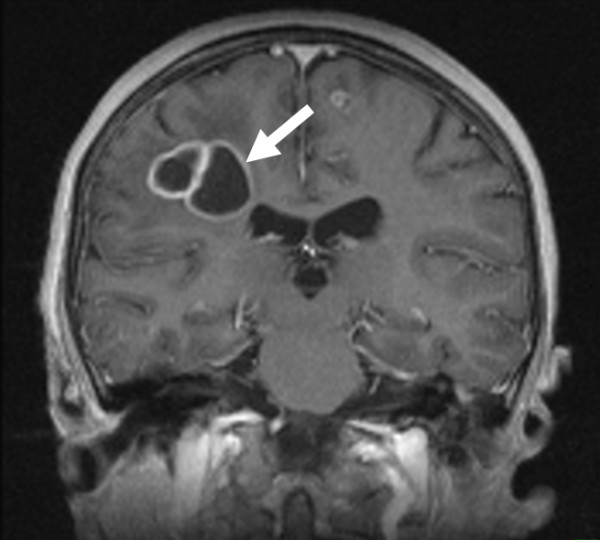
**Contrast enhanced T1w MR Image shows the cranial abscesses in the right fronto-parietal white matter with the surrounding edema (→) and a further small abscess in the left gyrus frontalis superior**.

To rule out the neurological deficits an MRI of the brain was performed, which indeed showed multiple abscess-typical, circular contrast-enhancing supratentorial lesions on both sides of the brain and even more lesions occipital and left frontal, which corresponded to the neurologic symptoms (Figure 4).

An aspiration or drainage was discussed, but because of the size and multiplicity of cerebral abscesses neurosurgeons favoured a non-invasive therapy.

We administered an intravenous antibiotic therapy with imipenem/cilastatin and amikacin for three weeks and later according to the susceptibility results we added trimethoprim-sulfamethoxazole (TMP-SMX) (good susceptibility of TMP-SMX, imipenem, meropenem and amikacin). Intravenous therapy was continued for six weeks and after discharge an oral trimethoprim-sulfamethoxazole therapy was administered for another twelve months.

Under pharmaceutical therapy and physiotherapy the neurological status improved and the patient was transferred to a neurologic rehabilitation three weeks after the operation.

In a follow-up cranial MRI six month later, the previously detected foci had become smaller or had completely disappeared.

## Conclusion

Because of its low incidence nocardia infections are not well known and are therefore very often not considered in the initial diagnosis.

We recommend keeping nocardia infection in mind for patients with atypical pneumonia unresponsive to empirical broad-spectrum antibiosis even in previously healthy patients, even more so if they have any suspicious tumour and/or neurologic symptoms.

Delay of adequate antibiotic therapy can have serious consequences.

Furthermore, this case shows the necessity to keep the possibility of an adrenal abscess in mind as one of the differential diagnosis for adrenal incidentaloma in the presence of such clinical and diagnostical findings.

## Competing interests

The authors declare that they have no competing interests.

## Authors' contributions

MT, YKV and KAG managed the patient and reviewed the literature. CI performed the microbiological analysis, PS analyzed the radiologic findings. MT was the main writer of the manuscript. YKV, JRI and KAG moderated the manuscript. All authors read and approved the final manuscript.

## Pre-publication history

The pre-publication history for this paper can be accessed here:

http://www.biomedcentral.com/1471-2334/9/194/prepub
